# N Doping to ZnO Nanorods for Photoelectrochemical Water Splitting under Visible Light: Engineered Impurity Distribution and Terraced Band Structure

**DOI:** 10.1038/srep12925

**Published:** 2015-08-11

**Authors:** Meng Wang, Feng Ren, Jigang Zhou, Guangxu Cai, Li Cai, Yongfeng Hu, Dongniu Wang, Yichao Liu, Liejin Guo, Shaohua Shen

**Affiliations:** 1 International Research Centre for Renewable Energy & State Key Laboratory of Multiphase Flow in Power Engineering, Xi’an Jiaotong University, Shaanxi 710049, China; 2 School of Physics and Technology, Center for Ion Beam Application, Wuhan University, Wuhan 430072, P. R. China; 3 Canadian Light Sources Inc., 44 Innovation Boulevard, Saskatoon, S7N2V3, Canada

## Abstract

Solution-based ZnO nanorod arrays (NRAs) were modified with controlled N doping by an advanced ion implantation method, and were subsequently utilized as photoanodes for photoelectrochemical (PEC) water splitting under visible light irradiation. A gradient distribution of N dopants along the vertical direction of ZnO nanorods was realized. N doped ZnO NRAs displayed a markedly enhanced visible-light-driven PEC photocurrent density of ~160 μA/cm^2^ at 1.1 V *vs.* saturated calomel electrode (SCE), which was about 2 orders of magnitude higher than pristine ZnO NRAs. The gradiently distributed N dopants not only extended the optical absorption edges to visible light region, but also introduced terraced band structure. As a consequence, N gradient-doped ZnO NRAs can not only utilize the visible light irradiation but also efficiently drive photo-induced electron and hole transfer *via* the terraced band structure. The superior potential of ion implantation technique for creating gradient dopants distribution in host semiconductors will provide novel insights into doped photoelectrode materials for solar water splitting.

ZnO has been extensively investigated as an excellent photocatalyst and photoanode candidate for solar energy conversion^1^,^2^,^3^, due to its superior inherent properties, including non-toxicity, low cost and high electronic conductivity, *etc.*^4^,^5^,^6^,^7^,^8^,^9^. However, because of the wide band gap of ~3.37 eV, ZnO can only response to ultraviolet (UV) light (4% in the solar spectrum). Therefore, in order to obtain high photoelectrochemical (PEC) performances for solar water splitting over ZnO photoanodes, a critical concept in designing ZnO-based PEC cells is to sensitize ZnO photoanodes to visible light.

Effective and controlled doping with metal/non-metal elements is a common method to modifying the optoelectronic properties of wide-band-gap semiconductors^10^,^11^,^12^. One general doping technique is to introduce dopants during crystal growth via sol-gel or hydrothermal process^13^,^14^. However, the doped impurities tend to aggregate on the surface of the host materials during crystal growth, when the dopant concentration exceeds its solubility in the host crystal lattice^15^. Moreover, heavy doping would to some extent destroy the crystal lattice, and subsequently the functional nanostructures of host materials^16^,^17^. As a well known post-growth doping strategy, NH_3_ treatment is effective to incorporate N dopants into wide-band gap materials (*e.g.*, ZnO nanorods) with original morphology maintained^9^. However, the N dopant concentration and distribution within host materials cannot be effectively controlled and facilely tuned. Advanced ion implantation was regarded as an alternative strategy of post-growth doping to sensitize wide-band-gap semiconductors in visible light region, by effectively controlling the dopant concentration and distribution. Anpo and co-workers prepared various (*e.g.*, Cr, Fe, V, Ni, Mn) doped TiO_2_ photocatalysts by ion implantation method^18^,^19^, in which the functional nanostructures of the host TiO_2_ can be preserved after even heavy doping process. Under visible light irradiation (λ > 450 nm), Cr ion implanted TiO_2_ demonstrated excellent photocatalytic activity for decomposing NO into N_2_, O_2_ and N_2_O, whereas the original TiO_2_ did not demonstrate any photocatalytic ability.

Fast recombination rate of photo-induced electron-hole pairs is another main reason that severely restricts the PEC performances of semiconductor photoelectrodes^20^. Creating nanostructured heterojunction with a type-II terraced band alignment was widely utilized to favor charge separation, and hence benefiting photocatalytic performance^21^,^22^,^23^. However, the crystal lattice mismatch between two components of the heterojunction may introduce numerous interfacial defects, which would act as charge recombination centers^17^. To address this problem, Mao and coworkers demonstrated an impurity engineered nanostructure of ZnO:Al-ZnO:Ni with shallow Al donor levels doped ZnO core and intragap Ni impurities doped ZnO ultrathin overlayer. The overlayer was utilized to absorb visible light and the core transported visible light induced electrons, by which the optical absorption and charge migration processes were successfully decoupled^17^. In the following study, they designed a ZnO/ZnO:Cr nanorods/nanosheets isostructural nanojunction for PEC water splitting. In this engineered structure, the presence of intra-band gap states associated with Cr impurities increased optical absorption, and the nanorod morphology provided a direct pathway for transporting photo-induced electrons to the back contact^24^. Using a NH_3_ heat treatment method, Yang *et al.* successfully synthesized N doped ZnO NRAs, which showed optical absorption extended to ~550 nm while PEC activity under visible light of wavelength < 440 nm^9^. Such disparity between optical absorption and PEC activity in visible light region may be due to the poor separation ability of visible light excited electrons and holes in the N-doped ZnO NRAs prepared *via* a nitridation process in NH_3_ atmosphere. Recently, van de Krol and coworkers presented a W gradient-doped BiVO_4_ photoelectrodes with terraced band structure^21^. It was revealed that the inherent electric field introduced by the engineered band structure would provide additional driving force for the charge separation and migration, resulting in reduced recombination rate and hence greatly promoted photocurrent density. A photocurrent up to 1.1 mA/cm^2^ at 1.23 V *vs.* RHE was obtained for the W gradient-doped BiVO_4_ photoanode^21^. By introducing co-catalyst and double-junction Si solar cell, a further increased photocurrent density of ~4 mA/cm^2^ was obtained.

Motivated by these works, herein we presented an ion implantation strategy to fabricate a gradient homojunction structure in ZnO NRAs by spatially engineering the functional impurity distribution of N dopants. It was expected that N doping would introduce additional impurity levels within the forbidden band gap of ZnO and effectively extend its optical absorption edges. Meanwhile, the gradiently distributed N dopants along the vertical [001] direction of ZnO nanorods created a terraced band structure, which provided internal driving force for effective charge separation. As a result, significantly enhanced visible-light-driven PEC water splitting performance for N doped ZnO NRAs was achieved. Although this PEC performance is still low for practical application, we believe that such a post-growth doping strategy could open new avenues in search of high efficiency semiconducting materials for solar water splitting under visible light.

## Results

### Fabrication of N gradient-doped ZnO NRAs

**Figure 1:**
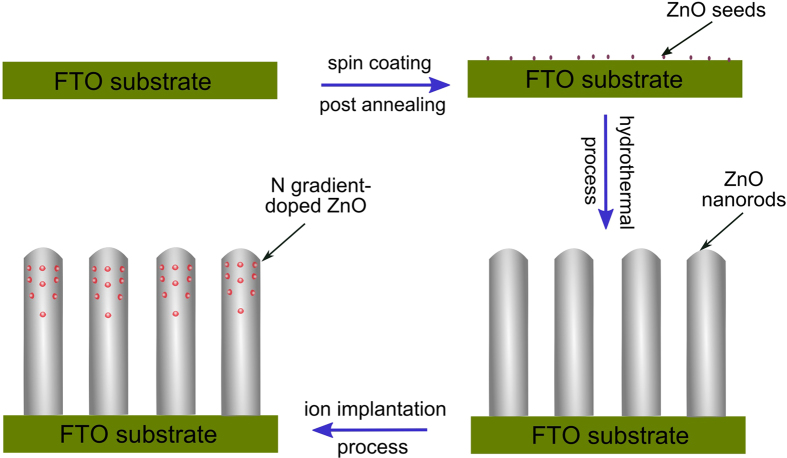
Schematic diagram of the preparation process of N gradient-doped ZnO NRAs by the advanced ion implantation method.

N gradient-doped ZnO NRAs were synthesized by a facile two-step method as schemed in Fig. 1. ZnO NRAs were firstly grown on the fluorine-doped tin oxide substrate (FTO, TEC-15, 15 Ω/sp) by a hydrothermal method and then doped with N element by an ion implantation process^25^. By tuning the implantation doses of N ions, the concentration of N dopants can be simply controlled. In this study, N doped ZnO NRAs with different implantation doses (1.25 × 10^14^, 2.5 × 10^14^, 5 × 10^14^, 1 × 10^15^ and 5 × 10^15^ ions/cm^2^) were named as N/ZnO–1, N/ZnO–2, N/ZnO–3, N/ZnO–4, and N/ZnO–5, respectively.

**Figure 2:**
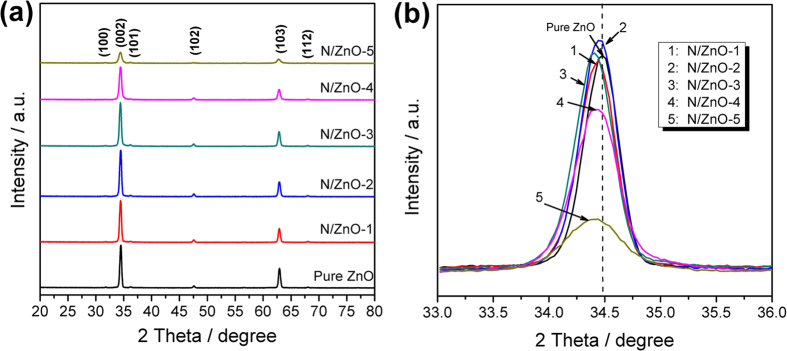
(**a**) XRD patterns of pristine ZnO and N doped ZnO NRAs with different implantation doses, (**b**) XRD patterns of the same samples as the diffraction angle changed from 33° to 37°.

Figure 2a shows the XRD patterns of N doped ZnO NRAs with different implantation doses as well as the pristine ZnO NRAs. The presence of sharp, narrow and well distinct peaks for all the samples provided the direct evidence for the crystallinity nature of as-prepared ZnO NRAs. The peak at 34.43°, corresponding to the (002) plane, is the most intensive peak in XRD patterns, which suggested that ZnO nanorods grew along [001] direction^7^,^26^. Additional peaks at 31.8°, 36.3°, 47.6°, 62.9° and 68.0°, which could be assigned to (100), (101), (102), (103) and (112) planes of wurtzite ZnO (reference code: 01-089-0510), respectively, were also observed, and no peaks related to any other impurity were detected for pristine or N doped ZnO NRAs. However, after the implantation of N ions, the diffraction peak intensity decreased distinctly. This may be due to the fact that the incorporation of N into ZnO reduced its crystallinity^27^. Figure 2b displays the detailed view of (002) patterns of the pristine and N doped ZnO NRAs. This peak position shifted towards lower angle, suggesting N ions were successfully doped into ZnO crystal lattice. As N atom has radius larger than O atom but smaller than Zn atom, the substitution of N at O sites or the insertion of N at interstitial sites in ZnO crystal lattice will increase the lattice distance^28^.

**Figure 3:**
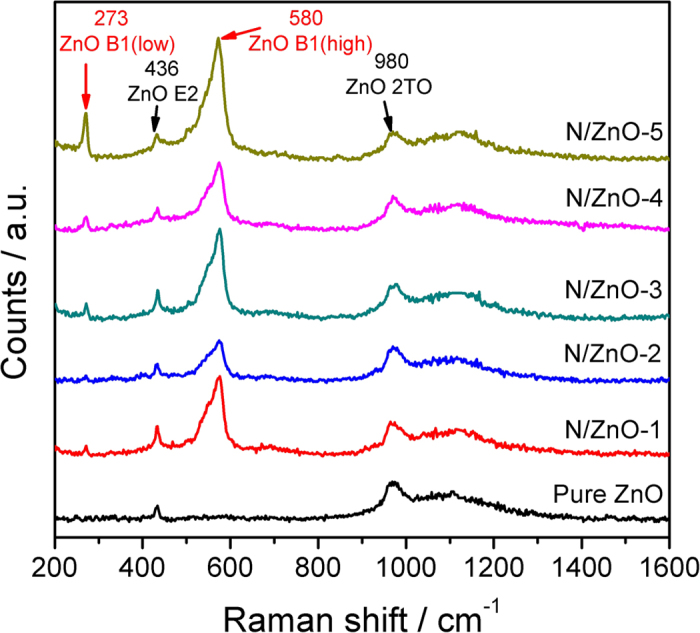
Raman spectra of pristine ZnO and N doped ZnO NRAs with different N dopant concentrations.

The crystal structure of N doped ZnO NRAs was further investigated by Raman scattering spectra, as illustrated in Fig. 3. Two inherent Raman bands at about 436 cm^−1^ and 980 cm^−1^, assigned to E2 (high) and 2TO (transverse-optical) vibration modes of wurtzite ZnO were observed^29^. Nevertheless, N doped ZnO NRAs displayed four distinctive Raman vibration modes centered at 273 cm^−1^, 436 cm^−1^, 580 cm^−1^, and 980 cm^−1^, respectively. Raman bands at 436 cm^−1^ and 980 cm^−1^ are the characteristic modes (E2 (high) and 2TO) for hexagonal ZnO, suggesting wurtzite ZnO structure was not destroyed by ion implantation. The other two bands at 273 cm^−1^ and 580 cm^−1^, could be attributed to electric field induced silent B1 (low) and B1 (high) modes, respectively^30^. It was believed that ion implantation process introduced disorder-activated Raman scattering (DARS) contributing to the emergence of silent B1 (low) and B1 (high) modes. Ion implantation process would create some defects and impurities in ZnO crystal, which destroyed the translational symmetry of ZnO and further induced the DARS^31^. Kaschner *et al.* assigned the Raman mode around 273 cm^−1^ to N related local vibrational mode (LVM), and used it to evaluate the relative dopant concentrations in N doped ZnO^32^. In the present study, no N related LVM was observed for pristine ZnO NRAs, while the N related LVM intensity increased gradually for the N doped samples with the increasing N implantation doses. This means N ions were successfully doped into ZnO crystal lattice, and the dopant concentrations could be effectively controlled by tuning ion implantation doses.

**Figure 4:**
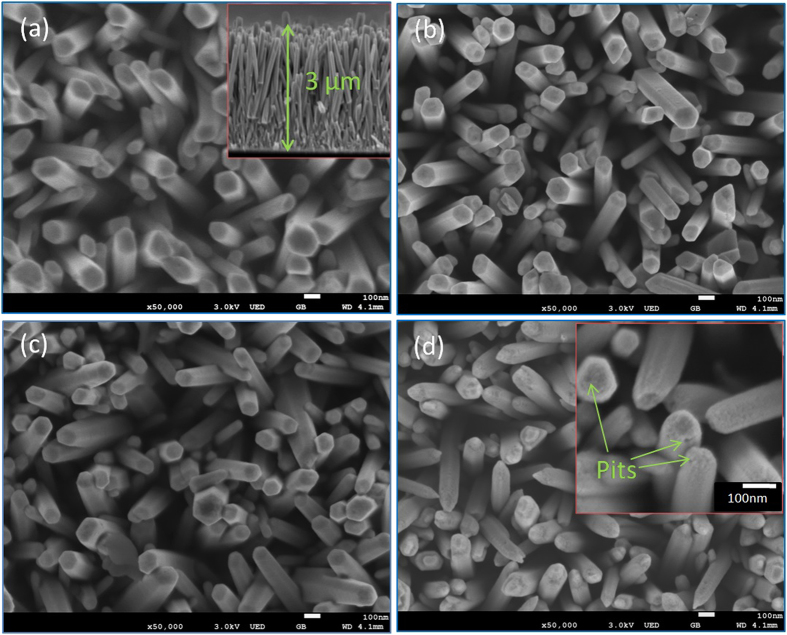
FESEM images of (**a**) pristine ZnO and N doped ZnO NRAs with different implantation doses: (**b**) N/ZnO-1 (1.25 × 10^14^ ions/cm^2^), (**c**) N/ZnO-3 (5 × 10^14^ ions/cm^2^), (**d**) N/ZnO-5 (5 × 10^15^ ions/cm^2^).

Figure 4 shows the SEM images of pristine ZnO and N doped ZnO NRAs (N/ZnO-1, N/ZnO-3, N/ZnO-5 as representative samples). One can see that vertical ZnO nanorods were hydrothermally grown and well dispersed on FTO substrate with diameter ranging from 60 nm to 120 nm and length of *ca.* 3 μm (Fig. 4a, inset). In addition, pristine ZnO NRAs showed smooth top and side surfaces. After N ion implantation, nanorod structure of ZnO was still maintained (Fig. 4b–d), suggesting ion implantation method would not destroy one-dimensional structure of ZnO NRAs after N doping. However, at high implantation dose (5 × 10^15^ ions/cm^2^ for N/ZnO-5), the top and side surfaces of ZnO nanorod became more rough, and some small pits could be observed (Fig. 4d, inset). During high ion dose implantation process, the surfaces of substrate materials could be to some extent etched, and some damages/vacancies could be introduced into ZnO. After post annealing treatment, these damages and vacancies were recovered and then aggregated into small pits.

**Figure 5:**
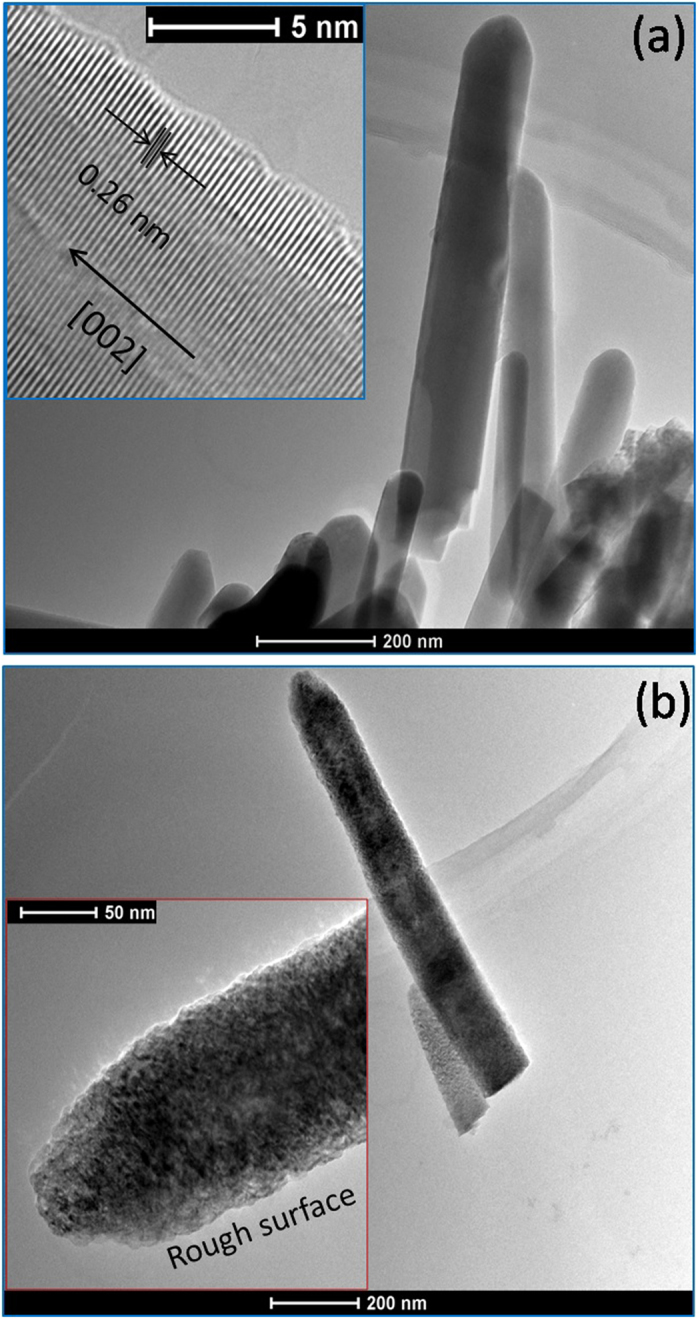
TEM images of (**a**) pristine ZnO and (**b**) N doped ZnO NRAs with the implantation dose of 5 × 10^15^ ions/cm^2^ (N/ZnO-5). The insets in (**a**,**b**) are the HRTEM images of pristine ZnO and N doped ZnO NRAs (N/ZnO-5), respectively.

Figure 5 shows the TEM images of pristine ZnO and N doped ZnO NRAs (N/ZnO-5 as the representative sample). The fringe spacing of pristine ZnO was *ca.* 0.26 nm (Fig. 5a, inset), corresponding to the (002) planes of hexagonal ZnO (reference code: 01-089-0510). The nanorod structure was not destroyed by ion implantation. While compared to pristine ZnO nanorod, N doped ZnO nanorods have rough surfaces, as etched by accelerated N ions during ion implantation. Any lattice fringe belong to zinc nitride was not found for N doped ZnO, maybe due to the high dispersion of N dopants in ZnO phase. Furthermore, the crystal lattice turned to be less-ordered for N doped ZnO nanorods, as previously evidenced by DARS in Raman spectra.

**Figure 6:**
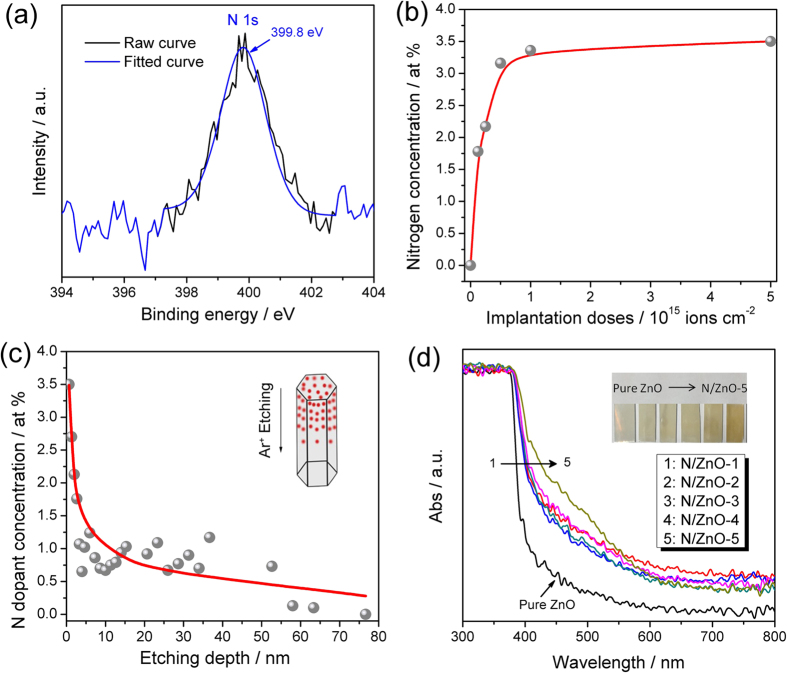
(**a**) N 1s XPS spectra for N doped ZnO NRAs with implantation dose at 5 × 10^15^ ions/cm^2^ (N/ZnO-5), (**b**) Atomic N concentration of N doped ZnO NRAs with different implantation doses, (**c**) XPS etching profile of N gradient-doped ZnO NRAs (N/ZnO-5) with implantation dose at 5 × 10^15^ ions/cm^2^, (**d**) UV-Vis spectra and digital photograph (inset) of N doped ZnO NRAs with pristine ZnO as the reference.

To study the chemical states of O, Zn, and N in N doped ZnO NRAs, systematic XPS analysis was performed, as illustrated in Fig. 6, S1 and S2 in Supplementary Information. From the survey scan in Fig. S1, XPS peaks belonging to O, Zn, N and adventitious C were observed, confirming the existence of N in ZnO after ion implantation. XPS Zn *2p* spectrum in Fig. S2a, displays the binding energies of Zn *2p*_3/2_ and Zn *2p*_1/2_ at 1021.3 eV and 1044.6 eV, confirming the formation of ZnO. As illustrated in Fig. S2b, the high resolution O *1s* spectra were deconvoluted into three binding energies: 531.5 eV, 529.6 eV and 529.1 eV, which could be assigned to the oxygen in water molecules absorbed on the ZnO surface^33^, the oxygen absorbed on the surface as –OH or O_2_, and the O atoms coordinated with Zn atoms^34^, respectively. It is well known that, depending on different synthesis methods, doped N ions in ZnO crystals always exist at two chemical states, *i.e.*, N_2_ molecules and N atoms occupying O sites^9^, with binding energies centered at about 404 eV and 396 ~ 397 eV, respectively. In Fig. 6a, only one peak centered at about 399.8 eV was observed. The absence of XPS peak (404 eV) assigned to N_2_ molecule suggested that N_2_ molecule was not incorporated in ZnO by ion implantation. Given the typical binding energy of N in zinc nitride (396 ~ 397 eV), the greater binding energy at 399.8 eV should be related to the formation of N-Zn-O bond^34^. As previously reported, when O atoms in O-Zn-O structure were partially replaced by N atoms, the electron density around N atoms decreased. Hence, it is reasonable that the N *1s* binding energy (399.8 eV, as here observed) in O-Zn-N bond is higher than that (396 ~ 397 eV, as previously reported) in N-Zn-N bond^34^.

The N atomic concentrations at the surface of N doped ZnO NRAs versus different implantation doses are illustrated in Fig. 6b. With the increase of implantation doses, N dopant concentrations increased gradually and then reached a plateau value of ~3.5%. This means that the impurity concentration could be effectively controlled by varying the implantation doses. Figure 6c displays the distribution of N dopants as a function of depth along the [001] direction for N doped ZnO NRAs by serial XPS etching measurements^35^. For N/ZnO-5, the atomic concentration of N dopants at top surface was about 3.5%, and then decreased to about 0.1% with the film etched for about 80 nm. According to the nature of ion implantation technique^36^, it is reasonable that the N atomic concentration decreased gradually with the increased depth. Thus, N doped ZnO NRAs with gradient impurity distribution along the vertical [001] direction of nanorods can be acquired from the ion implantation process.

Figure 6d displays the UV-Vis spectra of pristine ZnO and N doped ZnO NRAs. Pristine ZnO showed optical absorption only in UV region with the optical absorption edge of *ca.* 390 nm. After N ion implantation, the optical absorption edges of N doped ZnO NRAs showed significant red shift to *ca.* 550 nm. As shown in the inset of Fig. 6d, pristine ZnO film is white, whereas, after N ion implantation, all the films turn to be pale yellow or yellow, indicating their optical absorption ability in visible light.

### PEC performances of N gradient-doped ZnO NRAs

**Figure 7:**
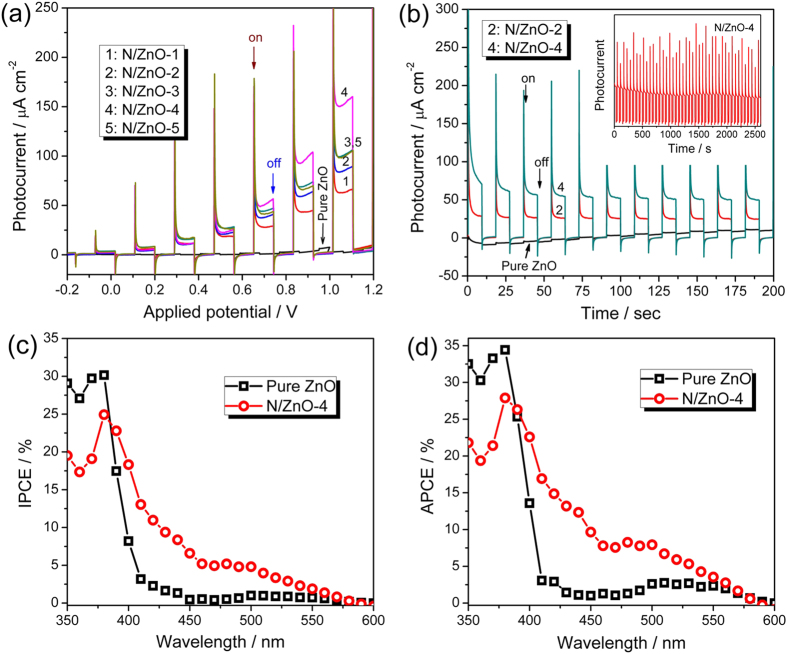
(**a**) Current-potential (I-V) plots of pristine ZnO and N gradient-doped ZnO NRAs under chopped light irradiation, (**b**) Time-dependent (I-t) curves of pristine ZnO, and N/ZnO-4 at an applied potential of 0.8 V *vs.* SCE, (**c**) IPCE and (**d**) APCE curves of pristine ZnO and N/ZnO-4 at an applied potential of 1.0 V *vs.* SCE. (Light source: solar light simulator with AM 1.5 filter (100 mW/cm^2^), electrolyte: 0.5 M Na_2_SO_4_ aqueous solution, counter electrode: Pt, reference electrode: SCE. A 420 nm cutoff filter was used in I-V and I-t measurements).

Figure 7a shows the I-V plots of pristine ZnO and N doped ZnO NRAs with different dopant concentrations under chopped visible light illumination (λ > 420 nm). For pristine ZnO NRAs, negligible photocurrent density was observed, whereas, all N doped ZnO NRAs showed considerable PEC performances under visible light. As the implantation doses increased from 1.25 × 10^14^ (N/ZnO-1) to 1 × 10^15^ ions/cm^2^ (N/ZnO-4), the photocurrent densities increased gradually, and then reached to a maximum photocurrent density of about 160 μA/cm^2^ for N/ZnO-4 at applied potential of 1.1 V *vs.* SCE. Further increasing the implantation dose to 5 × 10^15^ ions/cm^2^ resulted in a decrease in photocurrent density as observed for N/ZnO-5, which can be attributed to the high charge recombination rate as more defects were introduced by ion implantation at high doses.

I-t plots of pristine ZnO and N/ZnO-4 are shown in Fig. 7b. Again, the pristine ZnO NRAs showed limited PEC performance under visible light (λ > 420 nm), a negligible photocurrent density of 1.7 μA/cm^2^ was obtained at 0.8 V *vs.* SCE, mainly due to the poor optical absorption in visible light region. For N doped ZnO NRAs, considerable PEC activities were observed. At 0.8 V *vs.* SCE, N/ZnO-2 and N/ZnO-4 showed almost unchanged photocurrent densities of about 20 and 60 μA/cm^2^ during the 200-second measurement period. A long-term stability measurement further showed that the photocurrent density of N/ZnO-4 decreased slightly at the beginning and then stabilized in a 2600s I-t run (Fig. 7b, inset). As shown in Fig. 7a,b, the photocurrent curves demonstrated sharp anodic spikes after the light was switched on and then decayed to a steady photocurrent. It has been suggested that the spikes were the evidence of accumulated photoexcited holes at photoanode/electrolyte interfaces or the interfaces within electrodes^37^,^38^, and the accumulation of photoexcited holes could be mainly attributed to the existence of surface trap states^38^. As the gradient N doping could introduce structural defects (as shown in TEM images and Raman spectra) corresponding to surface trap states, the existence of anodic spikes were reasonable in this study. Further investigations to solve this problem of accumulation of photoexcited holes in the N gradient-doped ZnO NRAs are undergoing. For example, atomic layer deposition (ALD) process was considered to coat an overlayer (such as TiO_2_, Al_2_O_3_, *et al.*) to passivate the surface states of N gradient-doped ZnO^39^.

The incident photon to current efficiency (IPCE) and absorbed photon to current efficiency (APCE) measurements were conducted in the range of 350 nm to 600 nm (Fig. 7c,d), and displayed similar tendency. In the wavelength range of 390 nm to 550 nm, N gradient-doped ZnO NRAs (taking N/ZnO-4 as the representative sample) exhibited much higher IPCE and APCE values than the pristine ZnO NRAs. At 400 nm, the increased IPCE and APCE values of N gradient-doped ZnO NRAs (N/ZnO-4) were determined to be ~17.5% and ~22.5% for N/ZnO-4, respectively, suggesting N-doping induced visible-light response for ZnO. Moreover, when compared to the N doped ZnO NRAs prepared *via* a nitridation process in NH_3_ atmosphere, which only showed IPCE in the visible light of wavelength < 440 nm^9^, N gradient-doped ZnO NRAs encouragingly exhibited PEC activity (IPCE and APCE values) in visible light of up to 550 nm. It was further observed that more apparent difference existed between the APCE profiles than the IPCE profiles of pristine ZnO and N/ZnO-4 in the range of 400–550 nm, indicating N gradient-doped ZnO exhibited higher utilizable ratio of absorbed photons^40^.

**Figure 8:**
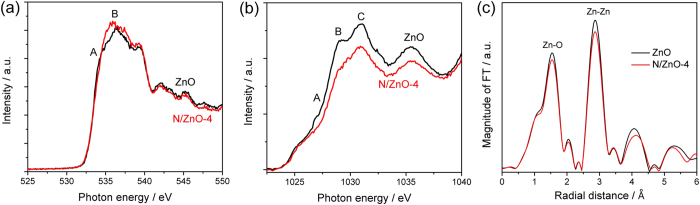
(**a**) O *K*-edge, (**b**) Zn *L*-edge XANES spectra, and (**c**) Fourier transformed k^3^χ data of Zn *K*-edge XANES of pristine and N gradient-doped ZnO NRAs (N/ZnO-4).

In order to understand more clearly the effects of N doping on the electronic structures and PEC performances of ZnO NRAs, X-ray absorption near edge spectra (XANES) and extended X-ray absorption fine spectra (EXAFS) were collected, as shown in Fig. 8. Owing to the dipole-transition selection rule, the features A-B of O *K*-edge spectra in Fig. 8a are related to the electron excitations from O *1s*-derived states to O *2p*-derived states; and given the fully occupied Zn *3d* states, the features A-C of Zn *L*-edge spectra in Fig. 8b are related to the electron excitations from *2p* electrons to unoccupied *4s* and *4d* states. Because the feature intensities are approximately proportional to the density of unoccupied states (UDOS)^41^, the obvious spectral intensity changes in both O *K*-edge and Zn *L*-edge spectra indicated the evident effects of N doping on the electronic structure of ZnO. The increased O peak intensity (*i.e.*, higher UDOS at O *2p* states) in Fig. 8a and decreased Zn peak intensity (*i.e.*, lower UDOS at Zn *4d* states) in Fig. 8b are attributed to higher Zn-O and Zn-N covalence, indicating that more electrons transfer from O *2p* states and N doping levels to Zn *4d* states, respectively. Moreover, it has been proved that both O *K*-edge and Zn *L*-edge XANES of ZnO are angle-dependent due to the existence of bilayer σ bond and *c*-axis oriented π bond^42^. Hence, the peak ratio change (B/A in O *K*-edge and B/C in Zn *L*-edge), regardless of the change of Zn-O bond angle or orientation, should be related to N substitution at O sites and/or doping into the interstitial sites, when the same measurement geometry is applied to ZnO and N doped ZnO. The Fourier-transformed (FT) k^3^χ data of Zn *K*-edge extended X-ray absorption fine spectra (EXAFS) of pristine and N doped ZnO NRAs are displayed in Fig. 8c. Notably, two main peaks were observed from FT k^3^χ spectra, which can be attributed to nearest neighbor (NN) Zn-O bond and next nearest neighbor (NNN) Zn–Zn bond, respectively^43^,^44^. Compared to pristine ZnO, N doped ZnO displayed decreased peak intensities for both Zn-O bond and Zn-Zn bond, suggesting deficient coordination number (possibly due to the distorted structure of O and Zn atoms^41^) induced by N doping *via* the ion implantation process, which has been evidenced by XRD and TEM measurements. All these observations convincingly support the successful N doping in ZnO crystal lattice by ion implantation process, which should contribute to the obvious optical absorption of N doped ZnO NRAs in visible light.

## Discussion

**Figure 9:**
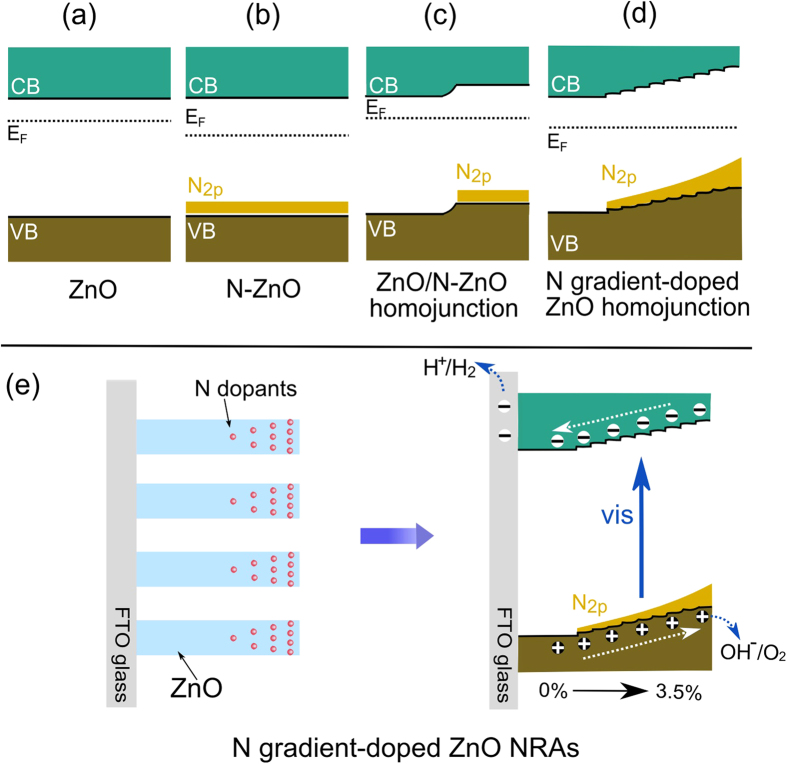
Energy band schematics of (**a**) pristine ZnO, (**b**) N doped ZnO, (**c**) ZnO/N-ZnO homojunction and (**d**) N gradient-doped ZnO homojunction, (**e**) Schematics of N gradient-doped ZnO NRAs and terraced band structure promoting charge separation.

It has been conceptually well-established in semiconductor physics that bulk charge separation can be efficiently enhanced by forming p–n0. homojunctions or homojunctions with gradient impurity composition. Recently, this concept was successfully adopted to design photoelectrode systems for solar water splitting. For example, Lin *et al.* created an n-p homojunction that could drive charge separation and produce an additional photovoltage by depositing a thin p-type layer of Mg-doped α-Fe_2_O_3_ over undoped n-type α-Fe_2_O_3_^45^. Abdi *et al.* demonstrated that the poor carrier-separation efficiency in BiVO_4_ photoanode can be overcome by introducing a gradient dopant concentration to develop a distributed n^+^–n homojunction^21^. Thus, in the present study, the enhanced charge separation in the N gradient-doped ZnO NRAs could be expected, given the gradient distribution of N dopants forming a homojunction with terraced band structure. The band structures of pristine ZnO, N doped ZnO and N gradient-doped ZnO were depicted in Fig. 9. Figure 9a shows the relative positions of conduction band (CB), valence band (VB) and Fermi level (E_F_) of the pristine ZnO. Generally, ZnO is an n-type semiconductor, and N doping will introduce acceptor levels above its VB, lowering the E_F_ of ZnO (Fig. 9b)^33^. Because of the difference of E_F_ between pristine ZnO and N doped ZnO, electrons will flow from pristine ZnO to N doped ZnO when they are brought into contact. Therefore, band bending would be induced by the alignment of E_F_ in the homojunction (Fig. 9c). For N gradient-doped ZnO, N dopant concentration at the top surface of ZnO nanorods was the highest (3.5%) and then decreased gradually with the increasing depth along ZnO nanorods. Terraced band structure was obtained accordingly in N gradient-doped ZnO homojunction (Fig. 9d), which was supposed to induce built-in electric field for promoted charge transfer. As schemed in Fig. 9e, when N gradient-doped ZnO NRAs were illuminated by visible light, the top surface region of ZnO NRAs will be excited by photons to produce electron-hole pairs. As driven by the terraced CB levels, the electrons at the CB of N doped ZnO NRAs can be efficiently transferred to the external circuit through the one dimensional pathway of ZnO NRAs for the reduction of H^+^ to H_2_. Meanwhile, the terraced VB levels induce internal driving force to facilitate the transfer of photo-induced holes to the top surface of ZnO NRAs. As a result, visible light induced charge carriers with poor mobility were efficiently separated^9^. In contrast to the heterojunction structure such as CdSe/TiO_2_^46^, CdTe/ZnO^47^, *et al.*, this N gradient-doped ZnO homojunction without obvious junction interface could yield lower concentrations of interfacial defects, and give rise to a considerable reduction in the recombination of electrons and holes excited by visible light^17^.

According to the systematic analysis on the relationship between physicochemical properties and PEC performances of N gradient-doped ZnO NRAs, it could be proposed that the PEC performance improvement in visible light should be attributed to the synergetic effect of narrowed band gap for visible light absorption and terraced band structure for promoted charge separation. After N doping, ZnO NRAs showed optical absorption edges extended to *ca.* 550 nm. Moreover, N doping did not change the rod-like nanostructure of ZnO NRAs, thus photogenerated electrons can be fluently transferred to the external circuit through the one-dimensional structure. As shown in Fig. 9, the impurity distribution induced alignment of the E_F_ in N gradient-doped ZnO NRAs led to the formation of terraced band structure and hence a built-in electric field, which effectively promoted the separation of photogenerated electrons and holes. As a result of the positive effects of gradiently distributed N dopants on the optical and electronic properties of ZnO NRAs, better visible-light-driven PEC water splitting performances over N gradient-doped ZnO NRAs were achieved. It was previously reported that N doped ZnO NRAs prepared by a NH_3_-nitridation process demonstrated optical absorption up to 700 nm, but only the visible light of wavelength < 440 nm could be utilized for PEC water splitting, indicating the visible light excited electron and hole pairs cannot be effectively separated^9^. For N gradient-doped ZnO in present study, visible light with wavelength up to 550 nm can be utilized for PEC water splitting (IPCE and APCE profiles), matching well with its optical absorption profile, which in turn experiment-evidently supported the charge carriers photoexicited by visible light could be efficiently separated, as driven by the N gradient-doping induced terraced band structure.

In summary, N ions were successfully doped into the hydrothermally grown ZnO NRAs by an ion implantation method. N doping did not change the rod-like morphology of ZnO NRAs, but extended the optical absorption edges to visible light region. Moreover, the gradient N dopant distribution led to a terraced band structure and hence a built-in electric field, contributing to the enhanced separation efficiency of photogenerated electron-hole pairs in ZnO. As a result, the visible light PEC performances for water splitting over N gradient-doped ZnO NRAs were improved significantly. When the implantation dose was 1 × 10^15^ ions/cm^2^, N doped ZnO NRAs achieved a maximum photocurrent density of ~160 μA/cm^2^ at 1.1 V *vs.* SCE, which was about 2 orders of magnitudes higher than the pristine ZnO NRAs. Although the PEC performance is still not high enough for the practical solar fuel conversion, the post doping approach for gradient distribution of foreign elements, which not only sensitized wide-band-gap semiconductor materials in visible light region but also created terraced band structure for promoted charge separation, provided the new insight for the development of novel semiconducting material for highly efficient solar water splitting, especially under visible light.

## Methods

### Fabrication of N doped ZnO NRAs

1. Firstly, the FTO glasses were ultrasonically cleaned in acetone, de-ionized water and ethanol for 30 min, successively, and then dried under N_2_ stream. The cleaned FTO glasses were spin coated by 0.1 M methanol solution of zinc acetate (Zn(CH_3_COOH)_2_), which was repeated for 8 times, and subsequently annealed at 350 °C for 30 min with a ramping rate of 5 °C/min. Then, the obtained ZnO seeded FTO glass was put into a mixed aqueous solution of zinc nitrate (Zn(NO_3_)_2_·6H_2_O) (0.05 M) and hexamethylenetetramine (C_6_H_12_N_4_) (0.05 M), which was maintained at 90 °C for 24 h. Finally, ZnO NRAs were acquired after annealing at 450 °C for 60 min with a ramping rate of 5 °C/min.
2. The ion implantation approach was performed by an ion-implanter consisting of a metal ion source, mass analyzer, high voltage ion accelerator, high vacuum pump and an appropriate substrate. This procedure was carried out for ZnO NRAs as substrates at room temperature with an accelerator voltage of 30 kV, and the nominal doses of N implantation were 1.25 × 10^14^, 2.5 × 10^14^, 5 × 10^14^, 1 × 10^15^ and 5 × 10^15^ ions/cm^2^, respectively. The N implanted ZnO samples were subsequently annealed at 450 °C for 60 min with a ramping rate of 5 °C/min.

### Characterization

The X-ray diffraction (XRD) patterns were obtained from a PANalytical X’pert MPD Pro diffractometer operated at 40 kV and 40 mA using Ni-filtered Cu Kα irradiation (Wavelength 1.5406 Å). The UV-Vis absorption spectra of the samples were determined with a Hitachi U-4100 UV-Vis near-IR spectrophotometer. The sample morphology was observed by a JEOL JSM-7800FE scanning electron microscope (SEM) and FEI Tecnai G2 F30 S-Twin transmission electron microscope (TEM) at an accelerating voltage of 300 kV. The chemical composition was obtained by X-ray photoelectron spectroscopy (Axis Ultra DLD, Kratos) with mono Aluminum Kα radiation. The charge calibration was done by correcting C1s line of adventitious carbon setting to 284.8 eV to compensate the charge effect. Raman scattering study was performed on a Jobin Yvon LabRAM HR spectrometer using an argon ion laser with 514.5 nm irradiation at 20 mW. The X-ray absorption near edge spectroscopy (XANES) spectra at the O *K*-edge and Zn *L*-edge were measured on the Spherical Grating Monochromator Beamline at the Canadian Light Source (CLS), a 2.9 GeV third generation synchrotron source; the Zn *K*-edge spectra were acquired at the IDEAS beamline of the CLS, using Ge(220) crystals and were recorded in fluorescence yield.

### Photoelectrochemical measurement

PEC measurements were carried out in a convenient three-electrode cell. The films of N doped ZnO NRAs as working electrodes were mounted onto a home designed electrode holder. The electrodes as exposed to a 0.5 M Na_2_SO_4_ aqueous solution were fixed at 0.785 cm^2^. A saturated calomel electrode (SCE) and a large area platinum (Pt) plate were used as the reference and counter electrode, respectively. An electrochemical workstation (chi760D) equipped with a 300 W Xe lamp solar simulator with adjustable power settings through an AM 1.5G filter (Oriel) (100 mW/cm^2^) were used for amperometric photocurrent–potential (I-V) and photocurrent-time (I-t) measurements, and a 420 nm cut-off filter used to prevent UV light from simulated solar light irradiation. IPCE (incident photon to current efficiency) measurements were performed using a solar simulator integrated with a computer-controlled monochromator (Newport 74126), a photochopper (PARC), and a lock-in amplifier (Signal Recovery) for photocurrent detection. The absolute intensity of the incident light from the monochromator was measured with a radiometer/photometer (International Light). All IPCE measurements were carried out under potential-controlled conditions, with 1.0 V as applied potential *vs.* SCE reference electrode. The IPCE and APCE (absorbed photon to current efficiency) values were calculated according to the formula as follows^48^:


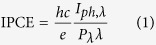



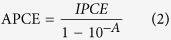


where *I*_*ph,λ*_ is the photocurrent density, *P*_*λ*_ is the power intensity of the incident light, *A* is the absorbance, and *h, c,* and *e* are Planck’s constant, speed of light in vacuum, and elementary charge, respectively.

## Additional Information

**How to cite this article**: Wang, M. *et al.* N Doping to ZnO Nanorods for Photoelectrochemical Water Splitting under Visible Light: Engineered Impurity Distribution and Terraced Band Structure. *Sci. Rep.* **5**, 12925; doi: 10.1038/srep12925 (2015).

## Supplementary Material

Supplementary Information
