# Supplementary material for: N Doping to ZnO Nanorods for Photoelectrochemical Water Splitting under Visible Light: Engineered Impurity Distribution and Terraced Band Structure
Source: Sci Rep. 2015 Aug 11;5:12925. doi: 10.1038/srep12925 (PMC4531782; doi:10.1038/srep12925)
Supplement: Supplementary Information [file srep12925-s1.pdf]

Supplementary Information

N Doping to ZnO Nanorods for Photoelectrochemical Water Splitting under Visible Light: Engineered Impurity Distribution and Terraced Band Structure

Meng Wang,[1] Feng Ren,[2] Jigang Zhou,[3] Guangxu Cai,[2] Li Cai,[1] Yongfeng Hu,[3] Dongniu Wang,[3] Yichao Liu,[2] Liejin Guo[1], Shaohua Shen[1]*

1. International Research Centre for Renewable Energy & State Key Laboratory of Multiphase Flow in Power Engineering, Xi'an Jiaotong University, Shaanxi 710049, China. Email: shshen_xjtu@mail.xjtu.edu.cn

2. School of Physics and Technology, Center for Ion Beam Application, Wuhan University, Wuhan 430072, P. R. China

3. Canadian Light Sources Inc., 44 Innovation Boulevard, Saskatoon, S7N2V3, Canada

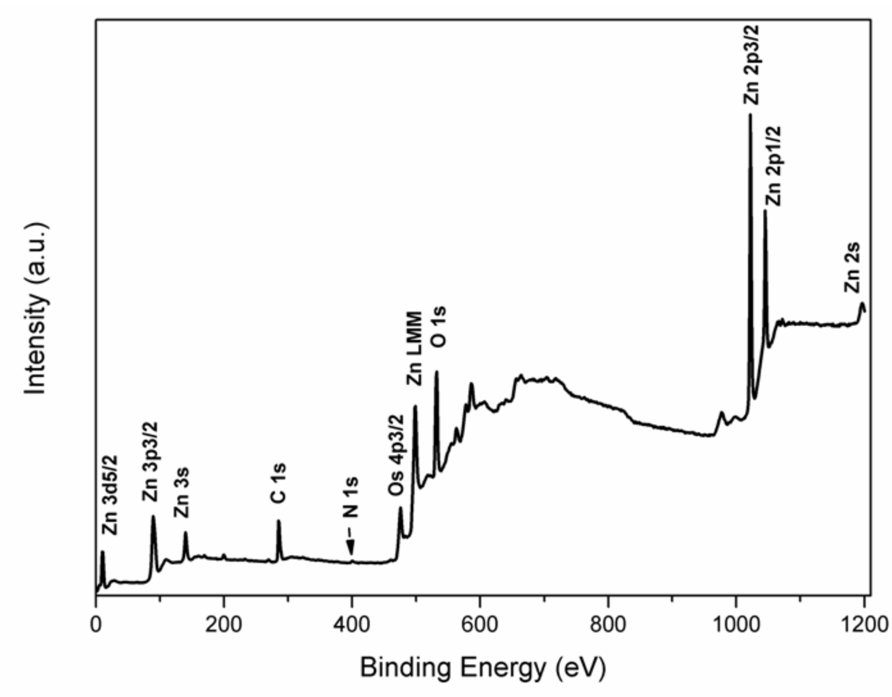

Fig. S1. Survey-scan XPS spectra of N gradient-doped ZnO NRAs at the implantation dose of 1×10^{15} ions/cm^2 (N/ZnO-4).

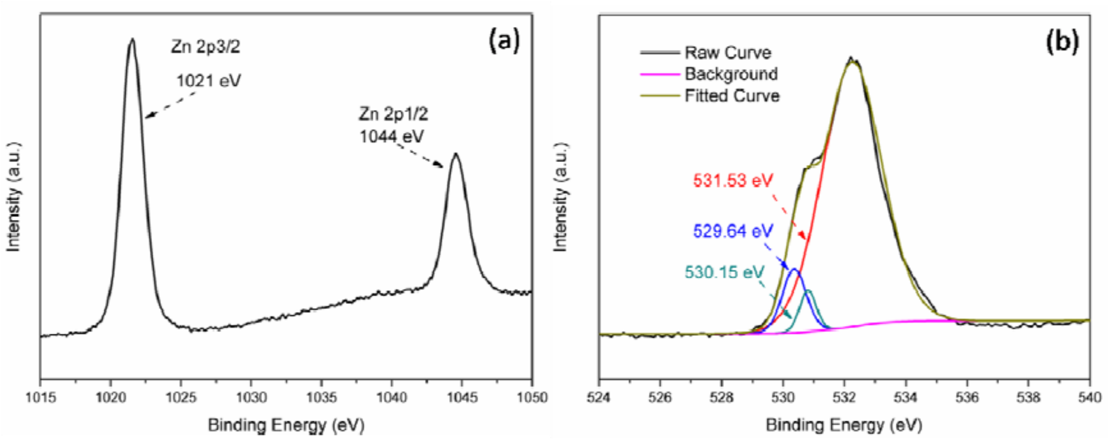

Fig. S2. (a) XPS Zn 2p spectra and (b) XPS O 1s spectra of N gradient-doped ZnO NRAs with implantation dose at 5×10^{15} ions/cm^2 (N/ZnO-5).
